# Gross Morphological Features of the Organ Surface Primo-Vascular System Revealed by Hemacolor Staining

**DOI:** 10.1155/2013/350815

**Published:** 2013-08-06

**Authors:** Chae Jeong Lim, Jong-Hyun Yoo, Yongbaek Kim, So Yeong Lee, Pan Dong Ryu

**Affiliations:** ^1^Laboratory of Veterinary Pharmacology, College of Veterinary Medicine and Research Institute for Veterinary Science, Seoul National University, Seoul 151-742, Republic of Korea; ^2^Laboratory Animal Research Center, College of Pharmacy, Hanyang University, Ansan 426-791, Republic of Korea; ^3^Laboratory of Clinical Pathology, College of Veterinary Medicine, Seoul National University, Seoul 151-742, Republic of Korea

## Abstract

The primo-vascular system (PVS), which consists of primo-vessels (PVs) and primo-nodes (PNs), is a novel thread-like structure identified in many animal species. Various observational methods have been used to clarify its anatomical properties. Here, we used Hemacolor staining to examine the gross morphology of organ-surface PVS in rats. We observed a sinus structure (20–50 **μ**m) with a remarkably low cellularity within PNs and PVs and several lines of ductules (3–5 **μ**m) filled with single cells or granules (*~*1 **μ**m) in PV. Both sinuses and ductules were linearly aligned along the longitudinal axis of the PVS. Such morphology of the PVS was further confirmed by acridine orange staining. In PN slices, there was a honeycomb-like structure containing the granules with pentagonal lumens (*~*10 **μ**m). Both PVs and PNs were densely filled with WBCs, RBCs, and putative mast cells (MCs), which were 90.3%, 5.9%, and 3.8% of the cell population, respectively. Granules in putative MCs showed spontaneous vibrating movements. In conclusion, the results show that Hemacolor, a simple and rapid staining system, can reveal the gross morphological features reported previously. Our findings may help to elucidate the structure and function of the PVS in normal and disease states in future studies.

## 1. Introduction

The primo-vascular system (PVS) is a novel anatomical network and new circulatory system. In the 1960s, Bong-Han Kim claimed that the PVS represented the meridians of acupuncture [1]. However, studies on the PVS have long been hampered because their isolation and identification have not been feasible. Recently, Dr. Soh's group developed several PVS-visualizing techniques and characterized the distribution, structures, and functions of the PVS [2, 3].

The PVS has been observed in small laboratory animals, such as mice, rats, and rabbits [4]. It has been consistently identified in various tissues including the surfaces of internal organs [5–8], blood vessels [9, 10], and lymphatic vessels [11] using special dyes, such as trypan blue [12, 13], acridine orange [9], and alcian blue [10, 11]. The organ-surface PVS has been primarily used in various PVS studies, as it is semitransparent, freely movable, and relatively easy to recognize with careful gross examination.

Based on various electron microscopic studies, Lee et al. [14] showed a bundle structure composed of several subducts (~10 *μ*m) and sinuses of various diameters in primo-vessels (PVs). Ogay et al. [15] reported follicle-like formations containing clusters of immune cells and several small channels or ductules (7−15 *μ*m) inside or near the formation in primo-nodes (PNs). Ogay et al. [15] also showed a bundle structure of several ductules (10−20 *μ*m) exhibiting characteristic rod-shaped nuclei in whole PVs and a tissue formation containing several lumens (6−10 *μ*m) in cross-sections of PVs.

At the cellular level, the PVS contains various types of immune cells, such as macrophages, mast cells (MCs), and eosinophils, which implies the system's potential role in immune responses [14–17]. In addition, PVS cells have been categorized into four major types based on current-voltage *(I-V)* relations recorded from the cells in PN slices [7]. Some PVS cells are much larger and rounder (10–20 *μ*m) with granules, whereas others are much smaller and round or appear similar to red blood cells (RBCs) [7, 8].

In previous studies, diverse staining methods and light and electron microscopy have been used to identify the PVS and characterize its structures [2]. For example, trypan blue has been used to identify the PVS in situ [12, 13]. Various DNA-specific staining dyes and confocal laser scanning microscopy (CLSM) analysis have been used to identify rod-shaped nuclei, the hallmark of PVs [2, 11]. Hematoxylin and eosin (H&E) staining has been mainly used for PVS cytology [15], whereas electron microscopy has been used for the ultrastructural characterization of the PVS (e.g., ductules, bundle structure) [14, 15]. However, trypan blue staining is limited in elucidating the cytology and anatomical structure of the PVS although it is simple to use. In addition, long processing times (about 24 hours) and/or sophisticated instruments like electron microscopes and CLSM are needed in the other methods. Thus, it would be desirable to determine rapid and simple methods to identify and morphologically characterize the PVS. In this study, we tested Hemacolor reagents, a rapid staining system used in hematology and clinical cytology [18, 19], to determine whether the staining can be suitable for PVS studies, in combination with a recently developed PVS-slice preparation method [7, 8].

## 2. Materials and Methods

### 2.1. Isolation of Organ-Surface PVS

Male Sprague-Dawley rats weighing 282 ± 13 g (*n* = 23; Orientbio Inc., Kyunggi-do, Korea) were housed in a temperature-controlled environment (20–26°C) with a relative humidity range of 40–70% under a 12 h light/dark cycle; they received water and standard rodent chow *ad *libitum. All animal experiments were carried out in accordance with the guidelines of the Laboratory Animal Care Advisory Committee of Seoul National University. The rats were anesthetized with an anesthetic cocktail (Zoletil, 25 mg/kg; xylazine, 10 mg/kg) administered by intramuscular injection. The abdomen of each rat was incised, and the PVS was sampled under a stereomicroscope from the surface of the abdominal organs, according to the methods reported previously [5, 12]. Briefly, we identified the organ-surface PVS tissue based on established standards: milky colored, semitransparent, and slightly flexible tissue composed of nodes and vessels.

### 2.2. PVS-Slice Preparation

We prepared the PVS slices according to the published protocol [7, 8]. Briefly, the intact PVS tissues that were isolated from the surface of internal organs were taken in a Ca^2+^-free Krebs solution supplied with O_2_ (95%)-CO_2_ (5%) and maintained in an ice-cooled Krebs solution (0–4°C). Meanwhile, 4% low-melting agarose (Lonza, Rockland, ME, USA) dissolved at 70°C was poured into a cubic frame (25 × 25 × 25 mm). When the agarose solution was chilled to 34–37°C, the PVS was embedded into the frame and then cooled on ice until the viscous solution was completely solidified. The agarose block was then taken out of the frame and firmly affixed to the bottom of a slicing chamber using instant glue before it was sectioned at a thickness of 200 *μ*m using vibrating microtome (1000 Plus, Vibratome, St. Louis, MO, USA). The resulting slices were incubated for 20–30 min in the oxygenated Krebs solution composed of (in mM) NaCl (120.35), NaHCO_3_ (15.5), glucose (11.5), KCl (5.9), CaCl_2_ (2.5), NaH_2_PO_4_ (1.2), and MgSO_4_ (1.2) followed by staining at 31°C [20].

### 2.3. Staining Methods for the Identification of PVS Cells

Hemacolor staining, a system of three solutions (solution 1, methanol fixative; solution 2, eosin stain; solution 3, methylene blue stain), was performed for the rapid cellular identification of PVS cell's WBCs and RBCs. The overall staining procedure of the PVS is as follows: either a PVS slice (200 *μ*m) or the whole PVS tissue was transferred into a drop of Hank's balanced salt solution (HBSS; Sigma, St. Louis, MO, USA) on slide glass and air-dried completely without water for 1–3 min. The slide glass was then dipped into and taken out of the solution 1 ten times for 10 sec. This staining process was repeated for solutions 2 and 3 and completed within 30 sec. Each stained PVS sample was kept in a drop of phosphate buffer solution (pH 7.2) for 20 sec, dipped into a distilled water three times for 10 sec, completely air-dried for 3–5 min, and then mounted with Canada balsam (Sigma). Using a stereomicroscope, low (100x and 200x) and high (1000x) magnification digital images were obtained from the Hemacolor-stained PVS cells. We took the pictures of the PVS tissues containing a micromeasure with a minimal unit of 0.01 mm and measured the luminal diameter of the tissues from the digital images. H&E staining, a method widely used for the morphological evaluation of various tissue types, was carried out to confirm the cellular composition of PVS cells and to determine their relative abundance. The PVS tissue was initially fixed overnight in 10% neutral buffered formalin, routinely processed, embedded in paraffin, and cross-sectioned at 3 *μ*m. The resulting PVS sections were stained with H&E as a part of the routine intake procedure. To identify DNA and RNA components in the PVS cells, each PVS sample was stained with 0.1% acridine orange solution for 15 min and then observed under a confocal laser scanning microscope (CLSM; LSM710, Carl Zeiss, Germany) in line with wavelengths of excitation and the emission of acridine orange [9, 21, 22]. To stain mast cells (MCs) in the PVS, the sample was stained with 1% toluidine blue solution for 3 min [23].

### 2.4. Mechanical Separation and Isolation of Single PVS Cells

For the cytological evaluation of the cellular component in the PVS, single PVS cells were prepared from intact PVS tissues as well as PVS slices on a slide glass by sprinkling the Krebs solution using a 1 mL syringe. Here, the motive power of isolating the PVS cells is solely the impact by the Krebs droplet, and trituration action was not applied to the PVS samples. The isolated cells were transferred to a slide glass followed by staining with Hemacolor in accordance with the procedure described above.

### 2.5. Digital Video Recording of Putative MC Movement of the PVS

One of the PVS slices in the incubation chamber was transferred to a recording chamber (0.7 mL) and was fixed with a grid of nylon threads supported by a donut-shaped silver wire weight while being perfused (3 mL/min) with oxygenated Krebs solution at 30–33°C [7, 8]. The movement of putative MC granules was observed by light microscope with differential interference contrast (BX50WI, Olympus, Tokyo, Japan) and recorded by a USB digital CCD camera series 150PIII.

### 2.6. PVS Cell Counting and Data Analysis

To determine the cellular composition of the PVS, individual PVS cells were counted from 25 rectangular fields (125 × 95 *μ*m) in the images of H&E-stained PVS slices at 1000x magnification. Caution is needed when selecting these sample areas because H&E-stained PVS slices (3 *μ*m in thickness) are very thin, and there may be some areas without cells in the edges of the slices. Considering this fact, we consistently avoided parts without cells in the PVS slices and selected only the fields filled with WBCs, RBCs, and putative MCs. Thus, we selected the representative PVS fields that showed uniform distribution of various cells. The sizes of PNs, PVs, and individual cells were measured using imageJ software (developed at the US National Institute of Health). All the data values were expressed as means ± standard errors, and the number of specimens or cells was represented by *n*.

## 3. Results

**Figure 1:**
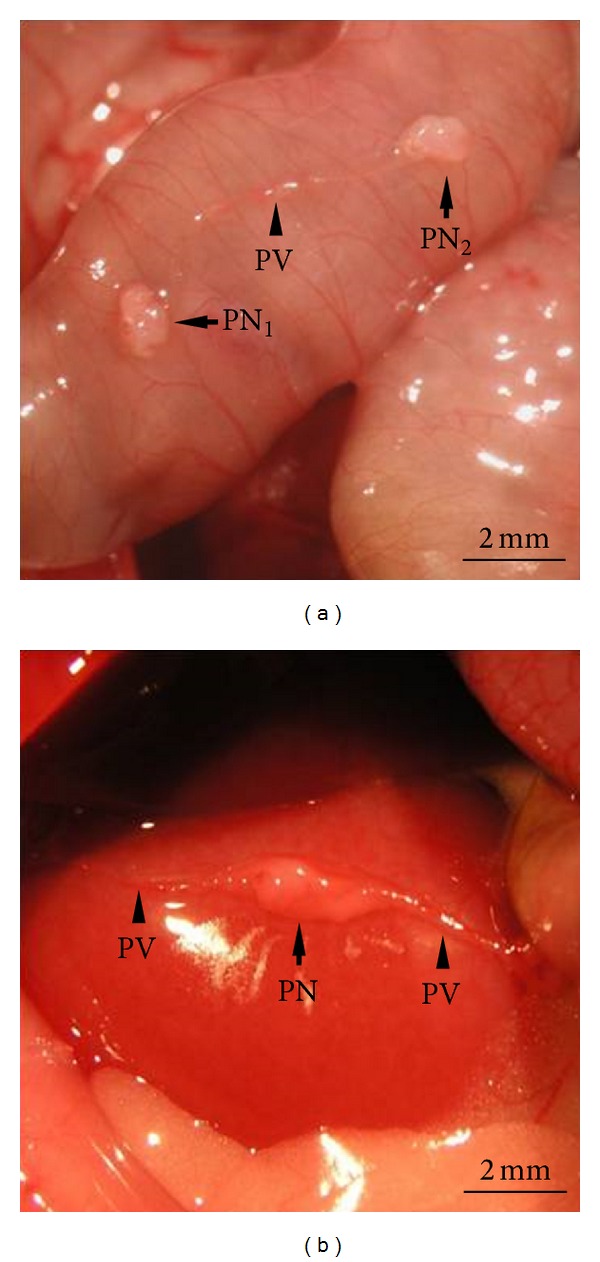
Intact PVS tissue identified on the surface of the abdominal organs in rat. (a) Representative example of a PVS tissue composed of two PNs (arrows) and a PV (arrowhead) on the surface of the small intestine (PN_1_, 1.22 × 0.86 mm; PN_2_, 1.17 × 0.77 mm; PV, 0.19 mm). (b) PVS tissue composed of an enlarged PN (arrow) and a typical PV (arrowheads) on the surface of the liver (PN, 2.57 × 1.11 mm; PV, 0.17 mm).

The results of this study were obtained from the evaluation of the 33 organ-surface PVS tissues from 23 rats. The PNs were collected mainly from the serosal surface of the small and large intestines (58.1%) and liver (35.5%) with or without PVs attached.  Figure 1(a) shows a representative PVS tissue on the surface of the small intestine composing of two PNs connected by a PV of typical size.  Figure 1(b) shows another example of PVS on the surface of the liver with an enlarged PN, which was even thicker than that of normal PNs. The average size of PNs was 1.26 ± 0.11 mm (major axis, 0.52–2.57 mm) and 0.73 ± 0.06 mm (minor axis, 0.34–1.50 mm, *n* = 27), and the average thickness of PVs was 0.25 ± 0.03 mm (*n* = 19). 

### 3.1. Hemacolor Staining of the Whole PVS

**Figure 2:**
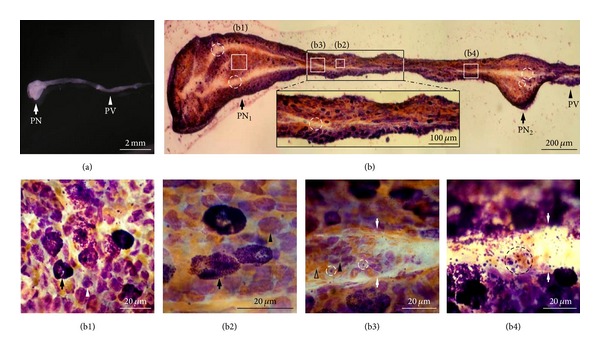
Images of the whole tissue and cells of the PVS stained by Hemacolor. (a) The unstained whole PVS sample in Krebs solution. (b) Typical unsectioned longitudinal image of a whole PVS tissue composed of PNs and a PV. Note the continuous inner space structures (dotted circles) along the longitudinal axis of the PVS. There are two spaces (dotted circles, PN_1_, 30–50 *μ*m; PN_2_, 10–50 *μ*m) in the PNs and one space (dotted circle in bottom inset, 20–30 *μ*m) in the PV. PVS cells at the edges were more abundant than in the middle of the PV. (b1) Distribution of the cells in the inner region (marked as “b1” in (b)) of the PNs. Note that most PN cells (arrow, large granular cells; arrowhead, small round cells) are round and are placed evenly and randomly. (b2) Distribution of the cells in the inner region (marked as “b2” in (b)) of the PV. Note that most PV cells (arrow, large granular cells; arrowhead, small round cells) are elliptical and horizontally arranged along the long axis of the PV. (b3 and b4) Distribution of the cells in the inner spaces (marked as “b3” and “b4” in (b)) of the PV. Note that the inner space (arrows) also contains numerous PVS cells (arrowhead, small round cells; open arrowhead, small yellowish cells; dotted circle, granules).

To visualize the cells in the PVS, we stained the PVS with Hemacolor, a rapid staining dye widely used in hematology and clinical cytology [18, 19]. In this study, PVS cells stained by Hemacolor refer to the cells within the inside of the walls of the cells, such as WBCs, RBCS, and MCs, and do not include the cells that compose the cell walls of the PVS.  Figure 2(a) shows a PVS sample isolated from the surface of internal organ in Krebs solution for staining.  Figure 2(b) is a representative stereoscopic image of the whole PVS stained with Hemacolor. The outer parts of the PNs and PVs were densely filled with cells, but the inner parts appearing as a white space (dotted circles in Figure 2(b)) were filled with little cells. The inner space showing low cellularity was continuous along the longitudinal axis of the PVS and had various luminal diameters depending on the location in the PVS. In general, the diameter of the space in PNs was larger than in PVs (30–50 versus 20–30 *μ*m), and there were two spaces in PNs (dotted circles of PN_1_ and PN_2_ in Figure 2(b)). The inner space could be identified by its different cellular composition and high number of granules (Figures 2(b3) and 2(b4)). Hemacolor-stained PVS cells were classified into the following three major groups based on their morphological properties: small round cells (majority), large granular cells, and small yellowish cells. The PN and PV cells differed, in that the PN cells were mostly round in shape and were distributed uniformly and randomly (Figure 2(b1)). However, most PV cells, including large granular cells and small round cells, were elliptical and were arranged in parallel along with longitudinal axes of the PVs (Figure 2(b)—bottom inset and Figure 2(b2)). In PVs, the staining properties of the three major cell types are similar to those in PNs. The major cells of the PVS were also located in the inner space within PVs (Figure 2(b3)). In particular, there were a number of granules (~1 *μ*m in diameter) in the inner space within PVs (Figure 2(b4)).

**Figure 3:**
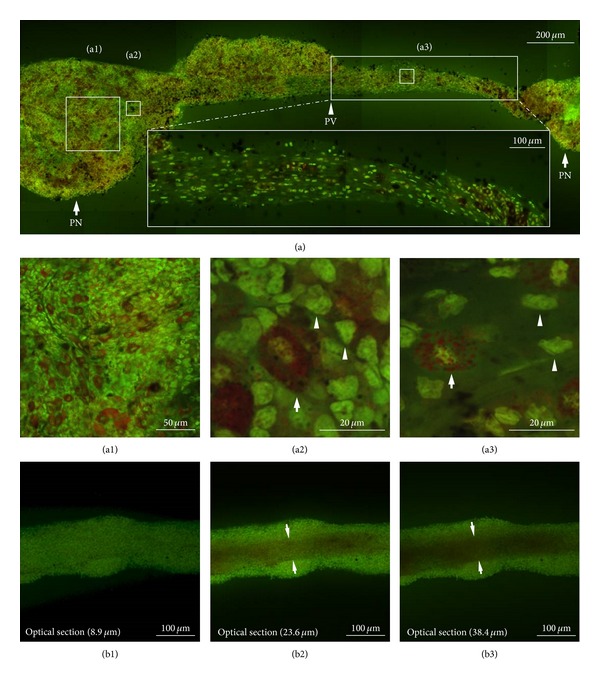
Confocal laser scanning microscopic images of whole PVS tissue and cells stained by acridine orange. (a) Unsectioned longitudinal image of a whole PVS composed of two PNs connected by a PV. Note that the tissue is densely filled with cells stained with green (majority) or dark brown. Some cells are linearly aligned along the longitudinal axis of the PV (bottom inset). (a1) PN cells with random distribution in the inner region (marked as “a1” in (a)). (a2 and a3) PN and PV cells at a high magnification (400x) (marked as “a2” and “a3” in (a)). Note the large cells (arrows) with granules stained red and small round cells (arrowheads) stained green in both the PNs and the PV. (b1, b2, and b3) Unsectioned longitudinal image of a whole PV showing an inner space according to depth of optical sectioning. Note that the inner space structure (arrows) devoid of cells is becoming darker with increasing depth of optical section.

To further confirm the morphological features of whole PVS tissue, we stained the tissue sample with acridine orange, which is DNA (green staining) and RNA- (red staining-) specific dye [21, 22], under a similar experimental condition to that in Figure 2. The cellular morphology observed in acridine orange-stained PVS tissue is similar to that obtained using Hemacolor staining. Most PV cells were stained green as shown in Figure 3(a) and were arranged in parallel along the longitudinal axis of the PV (Figure 3(a)—bottom inset and Figure 3(a3)), whereas PN cells were distributed randomly (Figures 3(a1) and 3(a2)). Most small round cells were revealed by their green color (denoting DNA) as a result of acridine orange staining, and large granular cells were revealed in green (denoting DNA) and red (denoting RNA) in nuclei and granules, respectively (Figures 3(a2) and 3(a3)) [21, 22]. The granules appeared dark brown at a low magnification (100x) (Figure 3(a)). As shown in Figure 2(b3) and Figure 2(b4), the inner space structure of the PV was also revealed by acridine orange staining according to depth of optical sectioning (Figure 3(b1–b3), see Movie S1 in supplementary material available online at http://dx.doi.org/10.1155/2013/350815).

**Figure 4:**
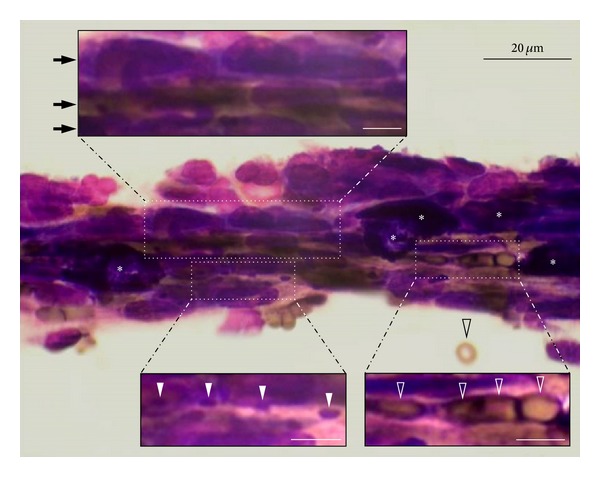
Longitudinal image of a thin PV stained with Hemacolor showing the multiple linear arrangement of cells (arrows in the top inset), small yellowish cells (open arrowheads in the bottom right inset), granules (arrowheads in the bottom left inset), and large granular cells (asterisks). Scale bars in the insets are 5 *μ*m.

Figure 4 shows a thin PV (30–40 *μ*m) stained by Hemacolor. As shown in Figure 2, various PVS cells (large granular cells, small round cells, and small yellowish cells) and granules are linearly aligned within along the longitudinal axis of the PV.

**Figure 5:**
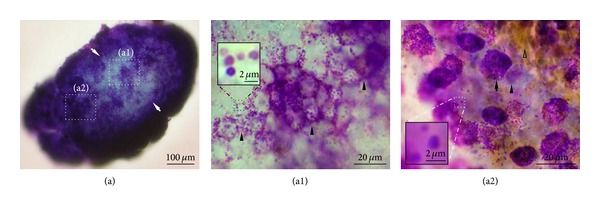
Honeycomb-like structure inside a PN slice (200 *μ*m) stained with Hemacolor. (a) Cross-sectional image of the PN-slice showing the inner space structure devoid of cells (arrows). (a1) The image of the inner space in the PN slice at a higher magnification. Note the honeycomb structure (arrowheads, about 10 *μ*m) and granules (inset) in the structure. (a2) The image of the outer part of PN slice stained with Hemacolor. Note the large granular cell (arrow), small round cell (arrowhead), small yellowish cell (open arrowhead), and the granules (inset).

To further characterize the morphology of the PVS, we stained a cross-section of a PN slice (200 *μ*m) with Hemacolor. In general, the cellular density is higher in the outer part and lower in the inner part of the PN slice as shown in Figure 5(a), which is similar to our findings in whole PVS tissue staining (Figure 2(b)). We observed a honeycomb-like structure in the inner space, and the diameter of the individual lumens in the honeycomb was ~10 *μ*m (Figure 5(a1)). In the honeycomb structure, granules were located within and on the borderline of each lumen. In addition, all three major cell groups were also found in the outer part of PN slices (Figure 5(a2)).

### 3.2. Cytomorphology of PVS Cells

**Figure 6:**
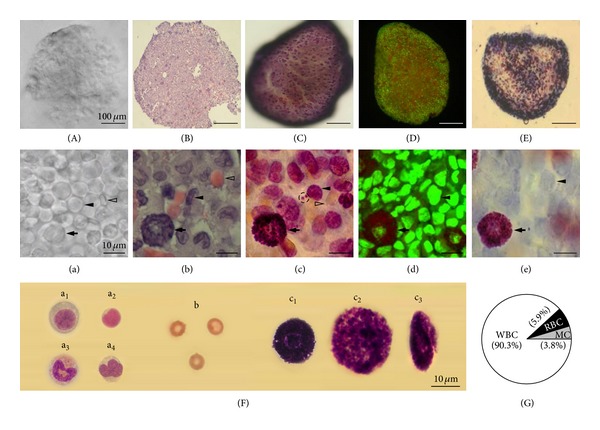
Three major groups of PVS cells revealed by various kinds of staining. (A and a) Unstained cross-sectional image of a PN slice with a thickness of 200 *μ*m. Note the PN cells resembling a large round cell (arrow, 12.69 *μ*m), a biconcave (flat)-shaped cell (open arrowhead, 7.42 *μ*m), and a small round cell (arrowhead, 9.42 *μ*m). (B–E) Typical cross-sectional images of the PN slice stained by H&E (B), Hemacolor (C), acridine orange (D), and toluidine blue (E). (b–e) Three groups of PN cells at a higher magnification displaying putative MCs (arrows), RBCs (open arrowheads), and WBC groups (arrowheads) which correspond to the neutrophils with lobulated nuclei. Note that the Hemacolor-stained PN slice clearly revealed the granules within putative MCs and isolated granules ((c), dotted circle). (D) and (d) are fluorescent microscopic images of acridine orange staining. (A)–(E) and (a)–(e) are images of different PVS tissues photographed at the same magnification. (F) Collection of three major groups of cells isolated from PVS tissue stained with Hemacolor. The individual PVS cells shown in ((F)-a_1_–a_4_) belong to the WBC group. WBC groups are composed of a plasma cell ((F)-a_1_)) with eccentrically placed nucleus, lymphocyte ((F)-a_2_)) with dense-staining nuclei and sparse cytoplasm, eosinophil ((F)-a_3_)) with eosinophilc cytoplasmic granules, and neutrophil ((F)-a_4_)) with multilobed nuclei and a lack of stained granules. The cells in ((F)-b) appeared in the group of normal mature RBCs. The cells shown in ((F)-c_1_–c_3_) are putative MCs (c_1_, typical (10–15 *μ*m); c_2,_ large (> 20 *μ*m); and c_3,_ elliptical type). (G) The cellular composition of the PVS. The PVS cells were counted from 25 fields (125 × 95 *μ*m) in images of H&E staining of PVS (*n* = 8) at 1000x magnification.

Figure 6(A) shows a stereoscopic image of an unstained PN slice at a low magnification. At a higher magnification of the unstained PN slices (Figure 6(a)), the PNs are densely filled with round cells of various sizes: large round (arrow, 12–20 *μ*m), biconcave (flat) disk-shaped (open arrowhead, 5–7 *μ*m), and small round cells (arrowhead, 8–10 *μ*m). Among these PVS cell groups, the small round cells were the most abundant. They were tightly packed like a cluster of grapes and evenly distributed in the area of the PN slices. Figures 6(B) and 6(b) illustrate the three groups of cells in the PN slice stained with H&E. In Figure 6(b), the large round cells were identified as putative MCs (arrow) based on their size and staining pattern in addition to their spherical nuclei and cytoplasm filled with intensely basophilic granules [24]. The small round cells stained dark blue and with round to horseshoe-shaped or multilobed nuclei (neutrophils, monocytes, and lymphocytes) were identified as WBC group (Figure 6(b), arrowhead). The biconcave-shaped cells without nucleus, yellow-stained cells by Hemacolor, were identified as RBCs (Figure 6(b), open arrowhead) [24, 25].

The three cell groups in the Hemacolor-stained PN slice (Figures 6(C) and 6(c)) had a staining pattern similar to those of H&E. In general, most PVS cells stained with Hemacolor were more clearly discernible than those stained with H&E under our experimental conditions. In particular, the images of putative MCs (Figure 6(c), arrow) and isolated granules (Figure 6(c), dotted circle) were more sharply visualized by Hemacolor staining. In the case of RBCs, however, H&E showed a staining quality superior to Hemacolor (Figure 6(b), open arrowhead).

Acridine orange staining also showed similar cytologic morphology of Hemacolor staining. The nuclei of the majority of WBCs stained by acridine orange were stained green (denoting DNA; Figure 6(d), arrowhead). In the putative MCs, the nuclei were stained green, whereas the granules were stained red (Figure 6(d), arrow), indicating the presence of DNA and RNA in nuclei and granules, respectively [21, 22]. Figures 6(E) and 6(e) illustrate the images of a PN slice stained with toluidine blue, which is known as a dye used to stain MCs [23]. The granules in these cells showed typical metachromatic staining, indicating that the large granular cells in the PVS were putative MCs. 

To further characterize the morphology of single PVS cells, individual cells were isolated from the tissues and stained by Hemacolor. We identified the WBC group of PVS, including the plasma cell (Figure 6(F), a_1_) with an eccentrically placed nucleus, lymphocyte (Figure 6(F), a_2_) with dense-staining nuclei and sparse cytoplasm, eosinophil (Figure 6(F), a_3_) with eosinophilic cytoplasmic granules, neutrophil (Figure 6(F), a_4_) with multilobed nuclei and a lack of stained granules, and RBC (Figure 6(F), b) with nonnucleated red-staining and typical size [24, 25]. Putative MC of PVS could be classified into typical (Figure 6(F), c_1_), large (Figure 6(F), c_2_), and elliptical types (Figure 5(F), c_3_) based on their morphological properties. Typical putative MCs had centrally placed nuclei and closely packed granules. As shown in Figure 2(b), the elliptical type of putative MCs was more abundantly distributed in PVs than in PNs.

The relative composition of the three groups of PVS cells, determined from the images of H&E staining (Figure 6(G)), indicated that the proportions of WBCs, RBCs, and putative MCs were 90.3%, 5.9%, and 3.8%, respectively. The average total number of the cells per PVS field (125 × 95 *μ*m) was 167.7 ± 4.12 (158–186) in eight PNs. The numbers of putative MCs, RBCs, and WBCs per field were 6.43 ± 0.89, 11.04 ± 3.23, and 148.81 ± 2.64, respectively. 

### 3.3. The Identification of Putative Mast Cells and Granules of PVS

**Figure 7:**
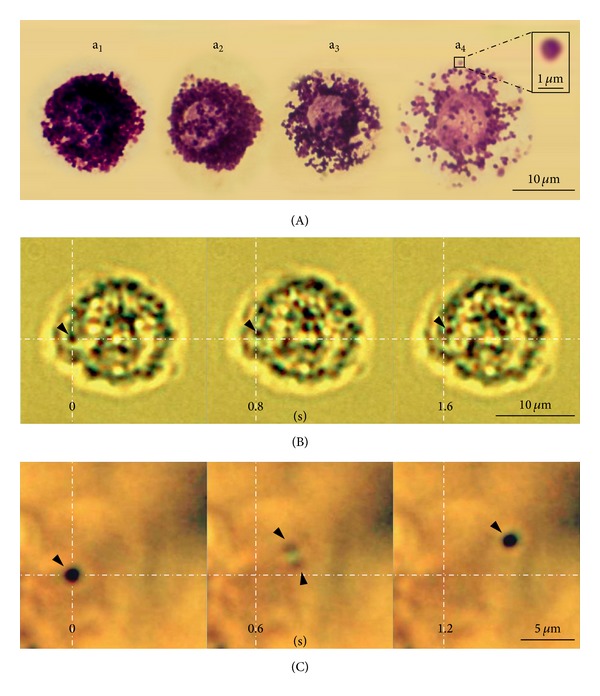
The properties of putative MCs of PVS and granules. (A) Classification of putative MCs of the PVS based on the degranulation condition. Note that the granules from a_1_ (typical) to a_4_ were increasingly degranulated; the granule size was about 1 *μ*m (inset of a_4_). (B) Continuous still image of granules in motion inside an MC. Note that arrowheads point to the granule that exhibits spontaneous vibrating movements on the right upper side. (C) Continuous still image of a granule with motility. Note that the granule displayed continuous vibrating movements and appeared as two divided granules for a moment (0.6 sec). This microscopy was performed on a live putative MC without any staining, which is described in the materials and methods section. The crossing points (B) and (C) of the two dotted lines indicate the location of the granule at *t* = 0. Selected frames from digital video recordings are presented.

In this study, putative MCs of the PVS contained granules of about 1 *μ*m (inset in Figure 7(A), a_4_). The degranulation stage of putative MCs differed from cell to cell (Figure 7(A), a_1_–a_4_). As shown in Figure 2(b4), the presence of typical putative MCs in the inner space of PVS was low, but degranulating putative MCs and isolated granules facing the inner space of the PVS were more abundant than in the outer area. We also observed that the granules in some putative MCs of the PVS had continuous and spontaneous movements. Degranulation of the putative MC in this study was not artificially triggered, and all occurred spontaneously. Figure 7(B) illustrates a representative still image of the granules in motion within a live putative MC (see Movie S2 in supplementary material available online at http://dx.doi.org/10.1155/2013/350815). In addition, we found that some of the isolated granules had spontaneous vibrating movements in random directions (Figure 7(C), see Movie S3 in supplementary material available online at http://dx.doi.org/10.1155/2013/350815). It is interesting that one granule appeared as two divided granules at one moment while moving (Figure 7(C)-0.6 sec). The average major and minor axes of recorded MCs with vibrating granules were 15.34 ± 1.45 (11.66–20.5 *μ*m) and 9.69 ± 1.01 (9.69–15.17, *n* = 9), respectively. The diameter of the putative MCs containing the granules in motion was also comparable to that of typical MCs stained with dyes as shown in Figure 6(F), c_1_.

## 4. Discussion

In this study, using Hemacolor staining, we confirmed the channel structures composed of a few sinuses (20–50 *μ*m) within PNs and PVs, and several lines of ductules (3–5 *μ*m) filled with single cells or granules (~1 *μ*m) in PVs. In a PN slice, there was a honeycomb-like structure containing granules with pentagonal lumens (~10 *μ*m). At the cellular level, the PVS was densely filled with WBCs (90.3%), RBCs (5.9%), and putative MCs (3.8%). Granules were also found within the putative MCs at various degranulation stages, and some granules showed spontaneous vibrating movements. The results of the present study indicate that Hemacolor is a promising staining system for the rapid identification and characterization of PVS cells and structures.

Hemacolor staining revealed that the PVS had an inner space structure with a lower cellular density. It is unlikely that this is an artifact formed by the slide glass suppressing the round tissue because the tissue was mounted with Canada balsam without pressing. In addition, the inner space was further confirmed by acridine orange staining of whole PV tissue (Figure 3). This inner space contained all three major cell groups of the PVS and granules (Figure 2). In some areas, RBCs and granules are the major contents of the inner space (Figures 2(b3) and 2(b4)). The inner space is similar to the “sinus” reported from previous studies using electron microscopy [14, 26], in that the sinus contained immune cells and granules. The present study newly reveals that the sinuses are continuous inner channels along the PVS that contain various PVS cells and granules. The detailed structures and functions of the sinuses in the PVS remain to be further studied. 

The most salient finding in this study is our characterization of the gross morphology of the PVS using Hemacolor staining. In general, Hemacolor staining has been used to stain and identify blood cells, such as lymphocytes, monocytes, and erythrocytes, in a short time period [18, 19]. We applied the Hemacolor staining method to the PVS for the first time and determined the most appropriate drying and staining times for the PVS. As a result, we were able to swiftly identify the cellular and structural features of the PVS. The major advantage of Hemacolor staining is that it takes just 5–10 minutes (drying time before staining: 1–3 min; Hemacolor staining for 30 sec; wash out for 30 sec; drying time after staining: 3–5 min) from the moment the whole PVS was sampled from the organ surface to the moment it was microscopically observed. In addition, by using Hemacolor staining in combination with PVS-slice preparation, we were able to identify the longitudinal part of the PVS, as well as its cross-section, within 30 min. The PVS staining method is faster and simpler than the H&E staining method, while maintaining good quality to allow the identification of the internal structure and the cellular morphology of the PVS. H&E staining, a common method to identify the PVS, takes about one day to microscopically observe the stained samples [2, 15]. Due to the short dying process involved in Hemacolor staining, we may have observed the PVS in a more natural state than that seen with previous methods. In addition, the application of Hemacolor on the PVS allows the major features of putative MCs and isolated granules in the PVS to be identified. Using Hemacolor staining, we confirmed all the previously reported immune cells stained with toluidine blue [23], H&E, and Wright Giemsa staining [14–16]. This method also allowed us to demonstrate the detailed features of the cells composing the PVS and revealed the sinuses within the PVS, ductules in PVs, and various individual cells in tissue or in isolation. Therefore, the Hemacolor staining method, combined with slice preparation, is suitable for the study of the PVS due to its fast identification of the gross and cellular morphology of the PVS.

From Hemacolor staining of the whole PVS and PN slices, we found evidence for the presence of subducts known as “ductules” in previous studies [14, 15]. The linear alignment of single cells and granules along the longitudinal axes of the PVs in this study (Figure 2(b) and Figure 4) is in good agreement with the linearly aligned elliptical or elongated cells with rod-shaped nuclei in PVs [2, 5], which have been considered a hall mark for the identification of the PVS [2]. This observation also provides evidence supporting the notion that the PVS is a circulatory channel [2, 3].

One of the novel findings of this study is the honeycomb-like structure inside the PN slice with pentagonal lumens of about 10 *μ*n in the honeycomb (Figure 5(a1)). The size of each lumen of the honeycomb structure (~10 *μ*m) is comparable to that of the ductules (10 *μ*m or 7–15 *μ*m) reported in the PV [14, 15]. In terms of its size, it is likely that each lumen of the honeycomb-like structure inside the PN may function as a ductule, as reported previously [1, 14, 15], and the channels for the flow of single cells or granules, as shown in Figure 4. The honeycomb-like structure in this study is similar to findings from prior cryoscanning electronmicroscopic studies [14, 26] in that individual lumens are tightly arranged in close contact, but distinctly different in that the size of the lumens is larger (~10 *μ*m versus 1–5 *μ*m) and much more homogeneous than that in the previous studies [14, 26]. This discrepancy may arise from the differences in experimental conditions and/or the types of PVS tissue tested. Further research is needed to understand the honeycomb-like structure observed in this study and its relation with the ductules reported in previous studies [14, 26] as well as the channels for the alignment of single cells or granules shown in this study (Figure 4).

In this study, we classified PVS cells into three major groups on the basis of their cytologic morphology: WBCs, RBCs, and putative MCs comprising 90.3%, 5.9%, and 3.8% of the cell population, respectively. Our overall findings were similar to those of recent studies using H&E staining and electron microscopy that showed the presence of numerous immune cells in the PVS, such as MCs, macrophages, and neutrophils [14–17]. We attempted to observe the PVS cells in tissue as well as the single cells in isolation from PVS tissue for more decisive observation of PVS cells. Under our experimental conditions using PVS slice preparation [7, 8], we were able to directly apply Hemacolor staining to intact live PVS cells (Figure 6(a)). 

The WBCs in the PVS were similar to those of typical WBCs and myeloid precursors, which are composed of neutrophils, plasma cells, eosinophils, and lymphocytes (Figure 6(F)) [24, 25]. The WBCs were consistent with the small round cells, which were further categorized into four types based on their current-voltage *(I-V)* relations recorded from cells in live PN slices in our previous electrophysiological study [7]. The presence of small clusters of RBCs in the PVS was previously reported [27]. In this study, we identified the RBCs in live PVS-slice preparation as well as in isolation as single RBCs after Hemacolor staining. The RBCs were similar to the normal mature rat RBCs in terms of the following properties: size (6–8 *μ*m), biconcave (flat) disk-shaped, nonnucleated cells with a central region of pallor appearing in middle of cytoplasm [24, 25]. The putative MCs in the PVS appeared most outstanding in the images of Hemacolor-stained PVS and were more densely populated at the edges than other parts of the PVS. We confirmed the putative MCs in terms of their morphology, such as their large cell body of purple color, typical metachromatic granules, and staining properties using toluidine blue, a dye commonly used for the staining of MCs [23]. Our observation is consistent with previous reports [14]. In the two types of rodent MCs, connective tissue and mucosal MCs [28], the putative MCs of PVS are similar to those in connective tissues because of their large size (12–20 *μ*m) and staining properties resulting from toluidine blue (Figure 6(e)). 

In this study, we recorded the movement of granules both within cells and/or in isolation. The fact that one can observe granule movement indicates that the physiological conditions for putative MCs [29] are reasonably well preserved under our experimental conditions in live PVS slices. In addition, the granules of putative MCs of the PVS and isolated granules are similar in morphology to the primo-microcells (Sanal), which are spherical or oval in shape and have a diameter of 1-2 *μ*m (Figure 7(A)) [2, 30, 31]. However, as the granules of the putative MCs and the primo-microcells were stained green (denoting RNA) and red (denoting DNA), respectively, by acridine orange, their components differed (Figures 3(a2) and 6(d)) [4, 32]. The critical issue of whether the granules in the putative MCs or in isolation in the PVS tissue are the primo-microcells still needs to be studied further. 

It is unusual for any tissue to have such a high proportion of immune cells as in the PVS: WBCs (90.3%), RBCs (5.9%), and putative MCs (3.8%). The present study is an attempt to determine the relative composition of the PVS cells. Recently, Kwon et al. [16] reported that the proportion of MCs is 20% of the whole immune cell population in the organ-surface PVS, which indicates that the proportion of MCs is different between the two studies, 20% versus 4.0%. The discrepancy may arise from the differences in the methods and experimental conditions and needs to be studied further. Since the cellular composition of the PVS is different from that of blood (RBC of over 90%), bone marrow (MC of 2.6% ± 0.5%), and spleen (majority of lymphocytes) [26, 33], such a unique cellular composition could be a useful hallmark for the identification and comparison of the PVS in future studies. 

In relation to the function of the PVS, the most salient point of the present observation is that Hemacolor staining of the PVS revealed a realistic integrated image of the PVS composed of the sinuses and the ductules reported in previous studies [14, 15]. The PVS sinuses (varying in size) and the ductules (~10 *μ*m) observed in this study are likely to function as circulatory pathways [2] since (1) the sinuses are channel-like structures throughout PNs and PVs, (2) the sinuses contain three major types of PVS cells and granules with random arrangements, and (3) the ductules are well developed in PVs and contain lineally aligned single cells or granules. Thus, the primary function of the PVS can be thought of as a pathway for the cells, such as putative MCs, WBCs, and RBCs. In this study, the immune cells, such as MCs, neutrophils, eosinophils, and lymphocytes, accounted for the vast majority of the whole PVS cell population (~94%). The results strongly support that PVS may have a crucial role in the initiation and/or maintenance of immunological functions [14–17]. We found the degranulation of putative MCs in the sinuses of PVs, indicating that the MCs were preferentially activated in the sinuses in PVs rather than in PNs (Figure 2(b4)). It is also known that MCs are rich at the acupoints [34–38], and the effects of acupuncture are related to MCs [34, 35]. Although both the PVS and the classical acupuncture meridian system are known to be associated with MC as described above, there is a lack of sufficient evidence to directly connect them in the current stage of research, and therefore additional research is necessary.

## 5. Conclusion

This study shows that Hemacolor staining is useful in identifying as well as in characterizing cellular and structural properties of the PVS by confirming typical morphological features of PVs and PNs with a simple light microscope in a short time period. Our results provide two pieces of morphological evidence supporting the circulatory nature of the PVS and its roles in relation to immune functioning. (1) There are two major channel structures in the PVS: sinuses and ductules. (2) The PVS is unique in its large population of immune cells, including a cellular composition of 90.3% WBCs, 5.9% RBCs, and 3.8% putative MCs. Of note, the MC population is high, and RBCs are present in PNs. These findings and the experimental approaches used in this study may help to elucidate the structure and function of the PVS in normal and disease states in future studies.

## Supplementary Material

Movie S1: Whole primo-vessel tissue was stained with acridine orange and the 3D microscopy was performed by a confocal laser scanning microscope (LSM710, Carl Zeiss, Germany) to show an inner space part within the primo-vessel. Most cells of the primo-vessel were stained green color (denoting DNA), and the inner space with low cellularity appeared dark along the longitudinal axis of the primo-vessel.
Movie S2: Spontaneous movement of granules within a putative mast cell in the primo-node slice was recorded under a light microscope with differential interference contrast (BX50WI, Olympus, Tokyo, Japan) using a USB digital CCD camera series 150PIII.
Movie S3: Spontaneous movement of a granule in isolation was recorded under a light microscope with differential interference contrast (BX50WI, Olympus, Tokyo, Japan) using a USB digital CCD camera series 150PIII.
